# Duplication appendiculaire révélé à l'occasion d'un syndrome appendiculaire récidivant

**DOI:** 10.11604/pamj.2015.20.335.6658

**Published:** 2015-04-08

**Authors:** Younes Aggouri, Pierlesky Elion Ossibi, Mourad Oussaid, Imane Tourghai, Karim Ibn Majdoub Hassani, Said Ait laalim, Khalid Mazaz

**Affiliations:** 1 Département de Chirurgie Générale (B), CHU Hassan II, Fès, Maroc

**Keywords:** Duplication, appendiculaire, laparotomie, Duplication, appendiceal, laparotomy

## Abstract

La duplication appendiculaire est une malformation très rare. Elle se rencontre chez les enfants exceptionnellement chez les adultes. Sa découverte est souvent fortuite à l'occasion d'une laparotomie ou laparoscopie pour une autre pathologie. Nous rapportons un cas particulier d'un patient qui a bénéficié d'une appendicectomie il y'a 3 mois qui est admis aux urgences pour un syndrome appendiculaire récidivant avec aux explorations radiologique et chirurgicale la présence d'une appendicite rétrocoecale.

## Introduction

Les duplications digestives (DD) sont des malformations congénitales rares. La duplication appendiculaire est exceptionnelle, avec une incidence déclarée de 0,004 [1, 2] C'est une affection qui se manifeste le plus souvent dans les premières années de vie, parfois certaines formes peuvent rester asymptomatiques et ne s'expriment qu’à l’âge adulte. Très peu d’étude ont été rapportées sur cette question. C'est ainsi que nous rapportons l'observation d'un patient qui a bénéficié d'une appendicectomie il y'a 3 mois qui est admis aux urgences pour un syndrome appendiculaire récidivant avec aux explorations radiologique et chirurgicale la présence d'une appendicite rétrocoecale.

## Patient et observation

**Figure 1:**
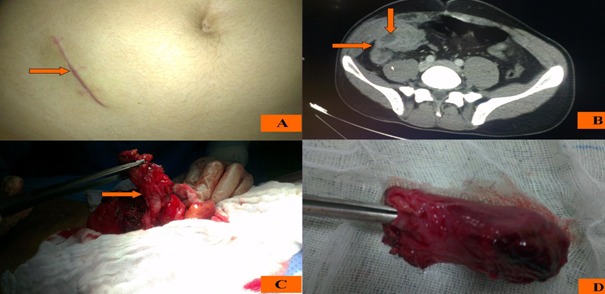
(A) incision de type Mc Burney; (B) image scannographique de l'appendice et de la collection abcédée; (C) image per opératoire de l'appendice; (D) pièce d'appendicectomie

Il s'agit d'un patient de 33 ans qui a bénéficié d'une appendicectomie il y'a 3 mois avec à l'exploration chirurgicale la présence d'un appendice phlegmoneux en position latéro-coecaleinterne et l'examen histologique de la pièce d'appendicectomie est revenue en faveur d'une appendicite aigue. L'histoire de la maladie actuelle remonte à 2 jours avant sa consultation par la survenue des douleurs de la fosse iliaque droite intenses évoluant dans un contexte de fièvre sans autres signes associés. L'examen clinique trouve un patient en bon état général, fébrile à 38°c avec à l'inspection une cicatrice de type Mc Burney (Figure 1A) et une défense à la palpation de la fosse iliaque droite. Le reste de l'examen clinique est sans particularité. Le bilan biologique montre un syndrome infectieux franc: hyperleucocytose à 19600/mm et C-réactive protéine à 186 mg/l. Le scanner abdominal objective un appendice rétro-coecal avec une collection abcédée en regard de l'incision (Figure 1B). Par ailleurs, il n'existe pas d’épanchement intra-péritonéal.

Notre patient a été mis sous antibiothérapie à large spectre. Une appendicectomie a été faite (Figure 1C, D). Les suites post opératoires sont simples. L'examen anatomo-pathologique de la pièce d'appendicectomie est revenu en faveur d'une appendicite aigue simple avec réaction inflammatoire péritonéale sans signe de malignité.

## Discussion

Les duplications appendiculaires sont des malformations congénitales rares et peu décrite dans la littérature. Moins de 100 cas ont été rapportés depuis sa première description en 1892 par Picolo [1–4]. Collins a signalé quatre cas (0,0008%) de l'agénésie congénitale dans une étude de 50 000 spécimens de l'annexe de vermiform humaine [5]. Cette affection se manifeste le plus souvent dans les premières années de vie, mais rarement à l’âge adulte. F. Calotã et al ont rapporté un cas survenant chez un patient de 45 ans [6] Les causes de cette anomalie n'est pas claire en raison de sa rareté et le manque de consensus d'opinions à ce sujet. Wallbridge a proposé une classification propre aux duplications appendiculaires permettant de distinguer trois types [7]: type A correspond à une duplication complète ou partielle dont seule la base est commune, les formes partielles sont moins fréquentes que les formes tubulaires complètes; Le type B est le plus fréquent (60%) et comporte deux sous groupes: le type B1 où les deux appendices sont disposés symétriquement par rapport à la valvule de Bauhin; Le type B2 où l'appendice est en position latéro-caecale habituelle et le second hypoplasique localisé sur une bandelette colique à distance plus ou moins grande du premier (Taenia coli type); Le type C correspond à une duplication caecale où chaque caecum est porteur d'un appendice propre. Le traitement est chirurgical par cœlioscopie ou a ciel ouvert, on réalisant une appendicectomie [6, 7].

## Conclusion

Bien que rare, la duplication appendiculaire est une anomalie malformative qui se rencontre à un âge précoce et exceptionnellement à l’âge adulte. Devant toute appendicite aigue, tout chirurgien doit bien examiner le caecum pour ne pas passer à côté d'une duplication appendiculaire.
